# Mapping and Cataloguing Microbial and Biochemical Determinants of Health: Towards a ‘Database of Salutogenic Potential’

**DOI:** 10.1111/1751-7915.70243

**Published:** 2025-10-01

**Authors:** Jake M. Robinson, Joel Brame, Christian Cando‐Dumancela, Sonali Deshmukh, Nicole W. Fickling, Scott Hawken, Claire Hayward, Emma Kuhn, Kevin Lee, Craig Liddicoat, Sunita Ramesh, Kate Robinson, Xin Sun, Martin F. Breed

**Affiliations:** ^1^ College of Science and Engineering Flinders University Bedford Park South Australia Australia; ^2^ The Aerobiome Innovation and Research Hub Flinders University Bedford Park South Australia Australia; ^3^ School of Biotechnology and Biomolecular Sciences University of New South Wales Kensington New South Wales Australia; ^4^ Environmental Health, College of Science and Engineering Flinders University Adelaide South Australia Australia; ^5^ School of Architecture and Civil Engineering The University of Adelaide Adelaide South Australia Australia; ^6^ Future Industries Institute University of South Australia Mawson Lakes South Australia Australia; ^7^ Department of Food Science and Microbiology, School of Science Auckland University of Technology East Auckland City New Zealand; ^8^ Zhejiang Key Laboratory of Urban Environmental Processes and Pollution Control CAS Haixi Industrial Technology Innovation Center in Beilun Ningbo China; ^9^ University of Chinese Academy of Sciences Beijing China; ^10^ State Key Laboratory of Regional and Urban Ecology, Ningbo Observation and Research Station, Fujian Key Laboratory of Watershed Ecology, Institute of Urban Environment Chinese Academy of Sciences Xiamen China

**Keywords:** aerobiome, AIR Hub, bioaerosols, biodiversity hypothesis, biophilic environments, old friends hypothesis, One Health framework, salutogenic

## Abstract

Microbial and biochemical research has historically focused on pathogenic agents due to their clear association with disease. This is a perspective that has saved countless lives but encourages a skewed, threat‐centered view of microbes and biogenic compounds. Emerging evidence shows that exposure to diverse environmental microbiomes and natural biochemical products is also salutogenic—promoting health and resilience. Here we introduce the ‘Database of Salutogenic Potential’, a *prototype* relational repository cataloguing environmental microbes and biochemical compounds linked to health benefits. Drawing from more than 200 articles, we identified 124 potentially salutogenic microbial taxa, 14 biochemical compounds and 63 associated benefits. By creating a structured and open platform, we aim to shift the balance between pathogen‐centric and salutogenic perspectives, potentially enabling future applications in public health, urban planning and ecosystem restoration. While the current iteration of the database primarily centers on human health outcomes, it is designed to expand into ecosystem health domains, embedding salutogenic thinking into One Health frameworks. We present this as a first step, not a ready‐to‐use tool, and invite collaborative refinement from the scientific community.

## Graphical abstract

We present a novel open‐access database cataloguing health‐promoting microbes and biogenic compounds. Designed to support research aligned with One Health and SDG3, this resource rebalances the traditional pathogen focus by consolidating data on salutogenic taxa, their benefits, and environmental origins—advancing holistic approaches to environmental and human health.

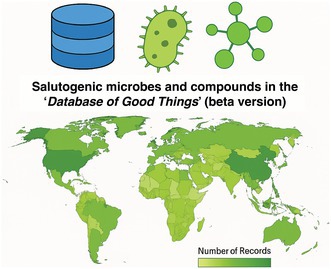

## 1 Beyond Pathogens

Microbial and biochemical research has long prioritized pathogens due to their undeniable impact on human, non‐human animal and plant health (Burger 1990; Kim et al. 2018). This emphasis on disease agents has undoubtedly saved countless lives. However, the result has been a skewed understanding of environmental microbes, biochemicals and their source systems, predominantly framed as threats rather than, or alongside, contributors to wellbeing (Robinson and Breed 2023). It creates a problematic ‘default position’ where the absence of any microbes and/or biological metabolites is considered most beneficial. This ‘threat‐based’ approach is a part of the post‐war hygiene paradigm.

Rather than viewing biodiversity as something to be eliminated, contemporary approaches recognise the vital role of diverse ecosystems in creating salutogenic (health‐promoting) environments. Salutogenic microbes—those that promote health—and beneficial biochemical compounds have received comparatively little attention despite their important roles in regulating immune function and metabolic processes, suppressing disease, mitigating stress and supporting ecosystem resilience (Li et al. 2022; Roslund et al. 2020; Robinson et al. 2024; Peddle et al. 2024).

Exposure to a diverse assemblage of microbes and organic biochemical compounds is vital for human and ecosystem health. For instance, Roslund et al. (2020) showed that a biodiversity intervention in early childhood education settings using local forest floor materials increased the diversity of the children's skin and gut microbiome (Roslund et al. 2020). In this study, exposure to naturally diverse microbiomes was also associated with enhanced immune function after 27 days. This and various other studies (Roslund et al. 2022) support the ‘old friends’ and biodiversity hypotheses, which posit that some modern non‐communicable health issues, such as allergies, autoimmune disorders and chronic inflammatory diseases, arise from a lack of exposure to co‐evolved microbes (e.g., soil microbes, helminths and other commensal organisms) that have historically played essential roles in calibrating the innate immune system (Rook et al. 2013). These ‘old friends’ help train the immune system to recognize and tolerate harmless antigens, ensuring proportionate responses and reducing the risk of overactivation (autoimmunity) or under‐activation (susceptibility to infection) (Rook et al. 2013). It has been proposed that industrialization, urbanization and reduced biodiversity have limited our exposure to these beneficial organisms, disrupting immune regulation (Haahtela 2019).

The science of ‘forest bathing’ or Shinrin yoku has shown that plant‐based biochemical compounds in the air can also have beneficial health effects. For instance, Li (2022) demonstrated that phytoncides, plant‐derived organic compounds (also known as ‘essential oils’), can have various beneficial impacts on human health, including enhancing natural killer (NK) cell activity, the number of NK cells, and the intracellular levels of anti‐cancer proteins, suggesting a preventive effect on cancers. Li and others' work (Lew and Fleming 2024) has also shown that phytoncides and forest bathing can reduce blood pressure, heart rate and stress hormones, such as urinary adrenaline, noradrenaline and salivary/serum cortisol and increase the levels of serum adiponectin and dehydroepiandrosterone sulphate, which have roles in regulating glucose levels and fatty acid breakdown. A relatively new concept called ‘aeronutrients’ is also worth considering in this environmental health research context—that the human respiratory system and olfactory pathways sequester airborne nutrients (vitamins, fatty acids, and trace minerals) that are beneficial for health (Fayet‐Moore and Robinson 2024).

In addition to the potential human benefits of exposure to salutogenic environmental microbes and compounds, we should consider the wider ecosystem health implications. For instance, Ryalls et al. (2022) show that flowers emit volatile compounds that are important for pollinators. Their research demonstrates that common air pollutants (e.g., nitrogen oxides and ozone) can react with these floral odours, reducing insect pollinator foraging efficiency. This example suggests that, in future database development, we should also consider salutogenic compounds in the context of non‐human animals and plants. Indeed, ‘One Health’ frameworks focus on the intersection of human, non‐human animal and environmental health (Destoumieux‐Garzón et al. 2018). Considering both pathogenic and salutogenic components of ecosystems in such frameworks will provide a more complete view of the One Health landscape.

In response to this historically understudied component of the exposome—and to promote a more holistic approach to environmental health—we introduce the ‘*Database of Salutogenic Potential*’: a relational and open prototype repository that catalogues and contextualises salutogenic microbes and biochemical compounds, including phytoncides and other potentially beneficial volatiles. This beta‐version tool is designed as a starting point: initially anthropocentric in scope, but explicitly expandable to non‐human and ecosystem contexts. Our aim is not to present a finished application but to provide a framework and invitation to co‐create a more balanced understanding of environmental determinants of health. The eventual aim will be to support researchers and practitioners in identifying potentially health‐promoting taxa or metabolites in environmental datasets that are worthy of further study. We have also developed the database architecture to incorporate information on the ‘pathogenic potential’ of microbes and compounds—acknowledging, for example, inverse U‐shaped dose–response relationships, immunocompromised host contexts, and the dynamic nature of pathobionts. Potential future applications may include the screening of candidate organisms for use in soil restoration or probiotic design, identifying bioactive volatiles in green infrastructure planning, and guiding exposure‐based interventions in clinical or urban settings. As a living database, it is designed to evolve through community contributions, emerging literature and interdisciplinary collaboration. Users can suggest new entries or database structural and content amendments by completing the Google Form in the Discussion.

## 2 Building the ‘Database of Salutogenic Potential’ (a Prototype)

**FIGURE 1:**
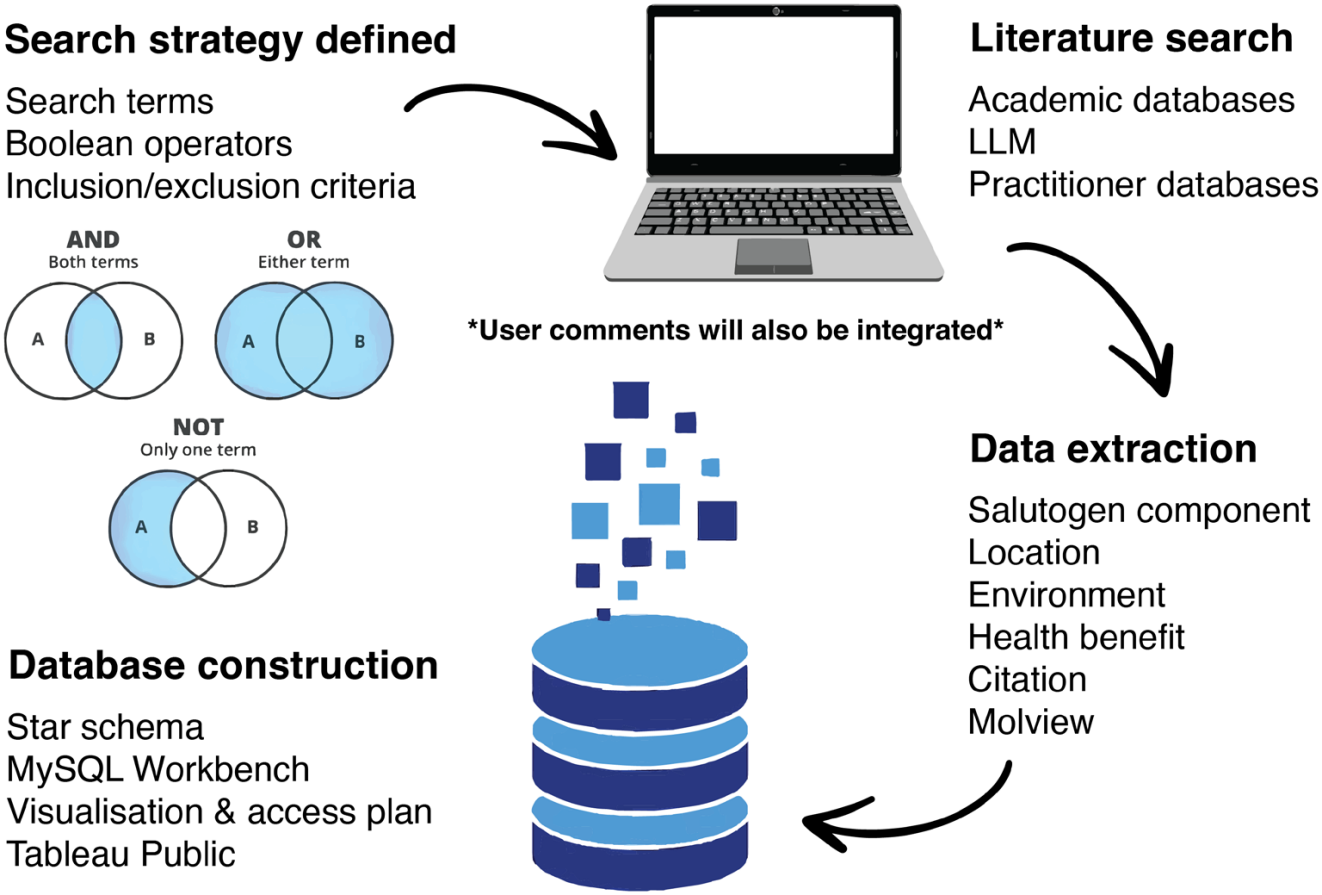
Workflow for identifying salutogenic components and constructing the relational database.

The beta version was assembled through a structured literature search across PubMed, Web of Science and Scopus (2000–2024). We focused on studies linking environmental microbes or biochemicals to beneficial human health outcomes, complemented by cross‐referencing existing resources such as the Optibac Probiotics Database. More than 300 articles were reviewed, with 238 meeting the inclusion criteria (Figure 1). See Supporting Information for the full methods.

**FIGURE 2:**
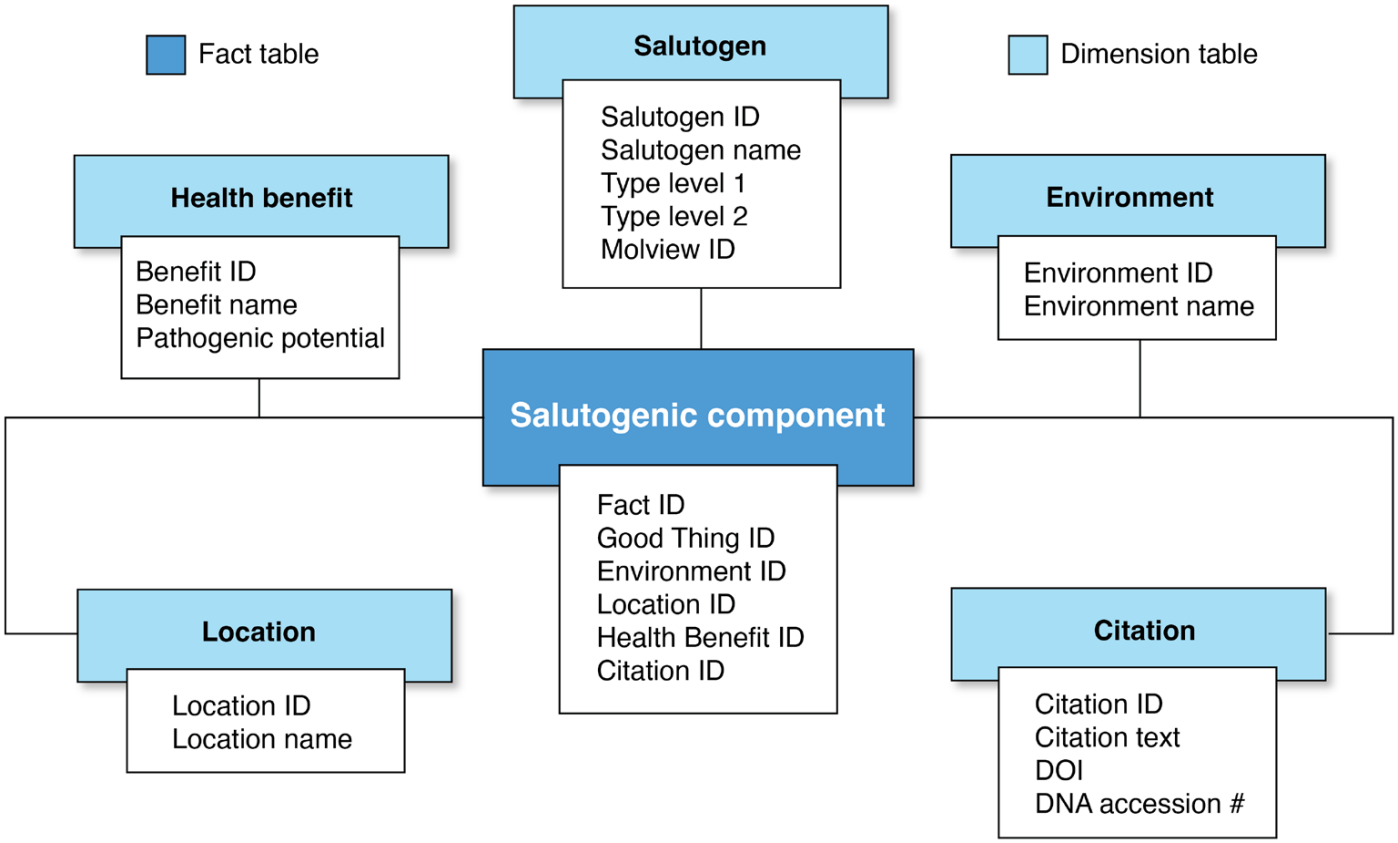
Database star schema, including the ‘Salutogenic component’ fact table and dimension tables (i.e., health effect [benefit and, where relevant, pathogenic potential], classification, location, environment, and citation). This schema illustrates the relational structure of the database rather than its downstream applications. Applications (e.g., in urban design, ecosystem restoration or clinical contexts) are enabled by, but conceptually external to, the schema and therefore not represented as a structural category here.

From this process, we identified 124 microbial taxa and 14 biochemical compounds with reported salutogenic associations, spanning 63 distinct health benefits (see Supporting Information for the full results). Examples include *Lactobacillus* and *Bifidobacterium* spp. (widely recognised for immune modulation in gut contexts), phytoncides such as α‐pinene and D‐limonene (linked to stress reduction and anti‐inflammatory effects), and compounds like geosmin, associated with positive affect and reduced depression‐like symptoms when inhaling the smell of healthy soil (Kim et al. 2025). The data were structured into a relational star schema (Figure 2), with a central fact table linking taxa or compounds to dimensions including health benefit, environment, and geographic occurrence. While technical detail is available in supplementary resources, the central point is that the schema enables queries such as: ‘Do my samples contain taxa associated with immune regulation?’ or ‘Which compounds contribute to stress reduction in forested environments?’

**FIGURE 3:**
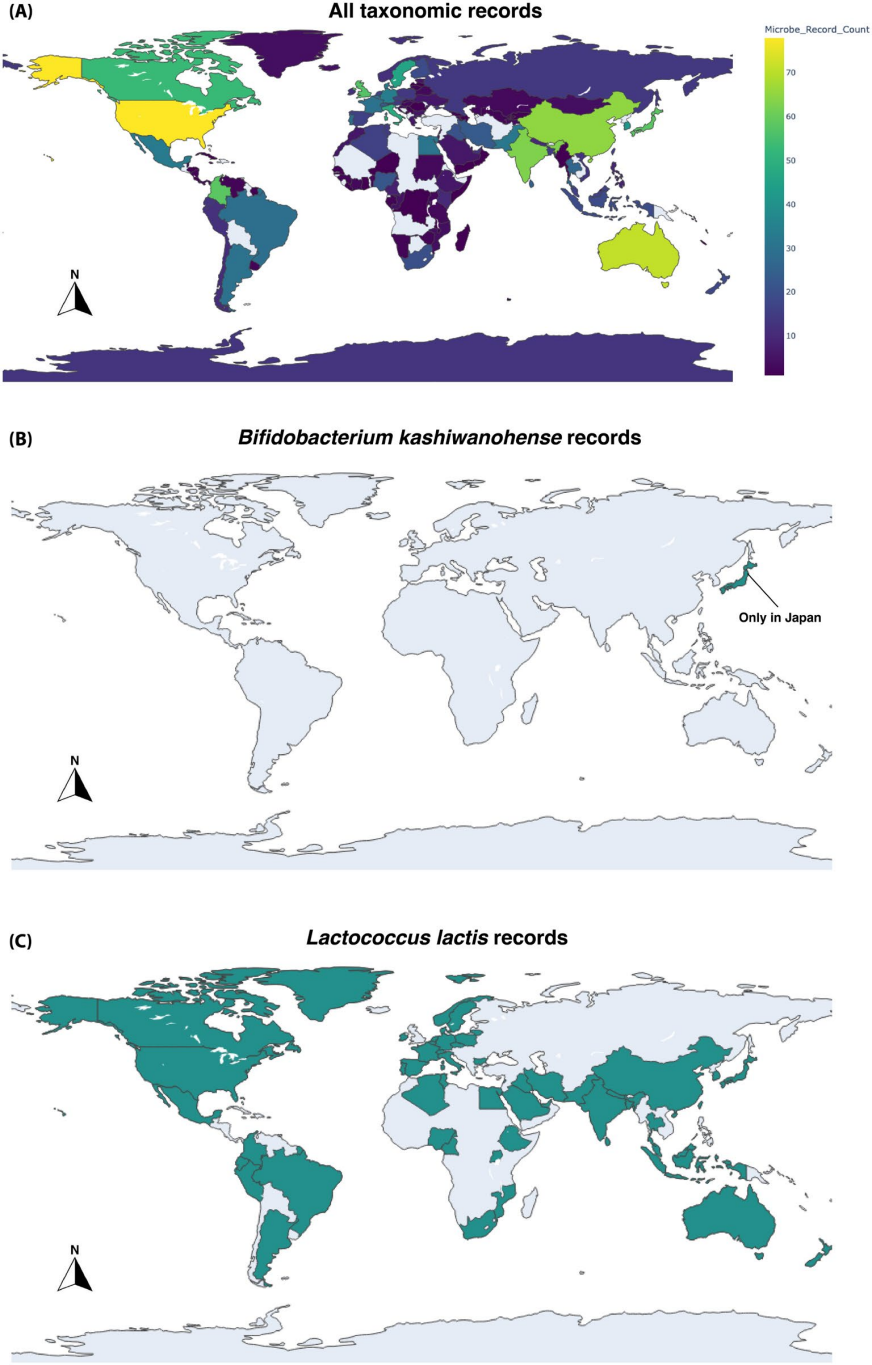
Example of a choropleth map showing (A) the number of distinct salutogenic microbial taxa recorded in each country, as reported in the Global Biodiversity Information Facility (GBIF), where colour intensity represents taxon richness, (B) the salutogenic microbe ( *Bifidobacterium kashiwanohense* ) with the lowest distribution of records, and (C) the salutogenic microbe ( *Lactococcus lactis* ) with the highest distribution of records. We have an R Shiny App (https://jakerobinson.shinyapps.io/salutogen_map‐1/), which will soon be connected to the database to allow users to create their own choropleth maps of salutogenic microbe records.

To encourage transparency and accessibility, the database was linked to Tableau Public for interactive exploration and supplemented with an R Shiny app mapping the biogeographic distribution of identified taxa via GBIF occurrence data (Figure 3). These visualisations are not intended as definitive maps of salutogenic potential—many microbes are globally distributed—but as proof of concept showing how occurrence data can be integrated and interrogated.

## 3 How This Version Should Be Viewed

We emphasise that this is a prototype, not a ready‐to‐use application. Therefore, several caveats apply:

- *Context dependence*: Microbes and compounds can confer benefits in one setting but risks in another. *Enterococcus* or *Clostridium* species, for example, have pathogenic potential in clinical contexts but also important ecological roles in decomposition. To reflect this, future versions will advance the ‘pathogenic potential’ dimension. Likewise, agents such as endotoxins cannot be classified as inherently salutogenic simply because of their role in immune training; they can also trigger harmful inflammatory responses depending on context (e.g., food contamination). This underscores the importance of framing categories relative to specific applications (e.g., environmental exposure vs. food safety), rather than assuming generalised ‘good’ or ‘bad’ classifications.
- *Application specificity*: What counts as salutogenic varies with context. For example, determinants for a salutogenic nursery may differ radically from those relevant to a tropical ecosystem restoration project. Applications will require tailored subsets of the database.
- *Completeness*: Current entries focus heavily on bacterial taxa and plant‐derived compounds; fungi (e.g., *Saccharomyces*) and actinobacteria (key antibiotic producers) remain underrepresented. Expansion is needed.
- *Evidence base*: Many reported benefits, such as stress reduction or improved mood, are based on small studies or limited biomarkers. Robust interventional trials and cross‐sector validation remain rare (Amato et al. 2021; Fujimura et al. 2014).

In other words, the database should be viewed as a scaffold for collaborative refinement, not as an authoritative list of ‘good microbes’.

## 4 Shifting the Pathogen‐Centric Paradigm

Despite its limitations, the Database of Salutogenic Potential opens new pathways. While pathogens pose clear threats to health, ecosystems also harbour salutogenic agents that support human health and wellbeing (Robinson and Breed 2023). Overlooking these components results in an incomplete picture of microbial and biochemical environments, the human exposome and determinants of health. Understanding the salutogenic potential of environments could inform public health interventions, ecosystem restoration efforts, urban planning, design and One Health approaches. Indeed, it is challenging to transform hygiene or sterile environmental design to consider materials and environments that promote salutogenic biomes rather than creating sterile spaces where threats may emerge unchecked by natural biodiversity. Cities and environments everywhere can benefit from increased knowledge of salutogenic microbes and biogenic compounds, and how we might better integrate them into our daily lives.

The One Health framework, which integrates human, (non‐human) animal and environmental health (Destoumieux‐Garzón et al. 2018), can also benefit from databases that highlight positive and negative microbial and biochemical interactions. After all, health is not ‘merely the absence of disease’ (Kühn and Rieger 2017). As such, One Health approaches should incorporate considerations for health *promotion* and *demotion*. Following further development, we envisage researchers querying environmental samples—such as soil, air, or water—against the *Database of Salutogenic Potential* to identify potentially salutogenic components. This will allow for a dual focus on such health‐promoting and demoting elements. Urban designers, landscape architects and environmental engineers could potentially use the information within this database, combined with representative surveys of environment types, land uses and management, to design green and blue spaces optimized for exposure to salutogenic microbes and compounds, supporting physical and mental well‐being. The database could also aid ecosystem restoration projects by identifying beneficial microbes and compounds to reintroduce into degraded ecosystems, enhancing soil health, biodiversity and ecosystem function. To this end, we aim to expand the database beyond its current anthropocentric focus to explore potentially salutogenic microbes and compounds for the wider ecosystem.

## 5 Challenges and Future Directions

A comprehensive characterisation of salutogenic agents is still in its infancy, requiring ongoing research to fill the many knowledge gaps. The effects of salutogenic microbes and compounds can also vary by environmental context and host factors, complicating generalisations. We plan to revise and expand database entries by incorporating emerging data (e.g., life stage dependencies) on salutogenic microbes and compounds as research progresses. We also plan to test these beta entries in real‐life projects prior to releasing a version that is user‐ready. There is also an opportunity for machine learning integration, that is, developing algorithms to predict salutogenic potential in uncharacterised samples based on database patterns. Further research on the pathogenic potential or other undesirable effects of an identified salutogen is also vital. For instance, a relationship characterised by an inverse U‐shaped dose–response curve may manifest—that is, too little or too much exposure may represent a health‐demoting relationship, whereas a moderate level of exposure may be optimal (Liddicoat et al. 2024; Calabrese et al. 2007). Such non‐linear hormetic effects and deficiency‐sufficiency‐toxicity relationships suggest that salutogens may offer health protection over an optimal dose range. Moreover, identifying ‘enablers’ and ‘antagonists’ will also be valuable. For instance, there may be mutualistic symbionts that have supportive effects on salutogens—and the same may apply to antagonists/inhibitors. As a living database, it is designed to evolve through community contributions, emerging literature and interdisciplinary collaboration. Users can suggest new entries or database amendments by completing the following Google Form: https://docs.google.com/forms/d/e/1FAIpQLSfBCE90ljYDlSuG0fkzxIaOwUEQbz0A2_2usl3jr9FyTWgBng/viewform?usp=sharing&ouid=100834116987245398655.

The ‘Database of Salutogenic Potential’ is presented here as a conceptual prototype intended to broaden perspectives in microbial and biochemical research. Rather than positioning it as a ready‐to‐use tool, we view this initial version as a foundation for iterative development—highlighting how salutogenic agents can be catalogued and explored alongside pathogenic ones. While the current focus is primarily on human health, future expansion will extend to ecosystem contexts, acknowledging the interdependence of human and ecosystem health. By signalling both the opportunities and the limitations at this early stage, we aim to catalyse dialogue, collaboration and refinement. Ultimately, with continued development and application, this initiative has the potential to shift how we conceptualise and harness the unseen determinants of health across diverse systems.

## Author Contributions

**Jake M. Robinson:** investigation, conceptualization, funding acquisition, writing – original draft, methodology, validation, visualization, writing – review and editing, software, formal analysis, project administration, data curation, supervision, resources. **Joel Brame:** writing – original draft, writing – review and editing. **Christian Cando‐Dumancela:** writing – review and editing, writing – original draft. **Sonali Deshmukh:** writing – original draft, writing – review and editing. **Nicole W. Fickling:** writing – review and editing, writing – original draft. **Scott Hawken:** writing – original draft, writing – review and editing. **Claire Hayward:** writing – review and editing, writing – original draft. **Emma Kuhn:** writing – original draft, writing – review and editing. **Kevin Lee:** writing – review and editing, writing – original draft. **Craig Liddicoat:** writing – original draft, writing – review and editing. **Sunita Ramesh:** writing – review and editing, writing – original draft. **Kate Robinson:** writing – original draft, writing – review and editing. **Xin Sun:** writing – review and editing, writing – original draft. **Martin F. Breed:** writing – original draft, writing – review and editing, supervision, funding acquisition.

## Ethics Statement

The authors have nothing to report.

## Conflicts of Interest

The authors declare no conflicts of interest.

## Supporting information


**Appendix S1:** mbt270243‐sup‐0001‐AppendixS1.zip.

## Data Availability

The data that support the findings of this study are available in Supporting Information of this article.
