# Supplementary material for: Mapping and Cataloguing Microbial and Biochemical Determinants of Health: Towards a ‘Database of Salutogenic Potential’
Source: Microb Biotechnol. 2025 Oct 1;18(10):e70243. doi: 10.1111/1751-7915.70243 (PMC12485223; doi:10.1111/1751-7915.70243)
Supplement: Supplementary file 1 — Appendix S1: mbt270243‐sup‐0001‐AppendixS1.zip. [file MBT2-18-e70243-s001.zip › mbt270243-sup-0005-DataS3.docx]

**R Shiny App**

Choropleth Map of Salutogenic Microbe GBIF Records

Top of Form

Bottom of Form

[**https://jakerobinson.shinyapps.io/salutogen_map-1/**](https://jakerobinson.shinyapps.io/salutogen_map-1/)

**
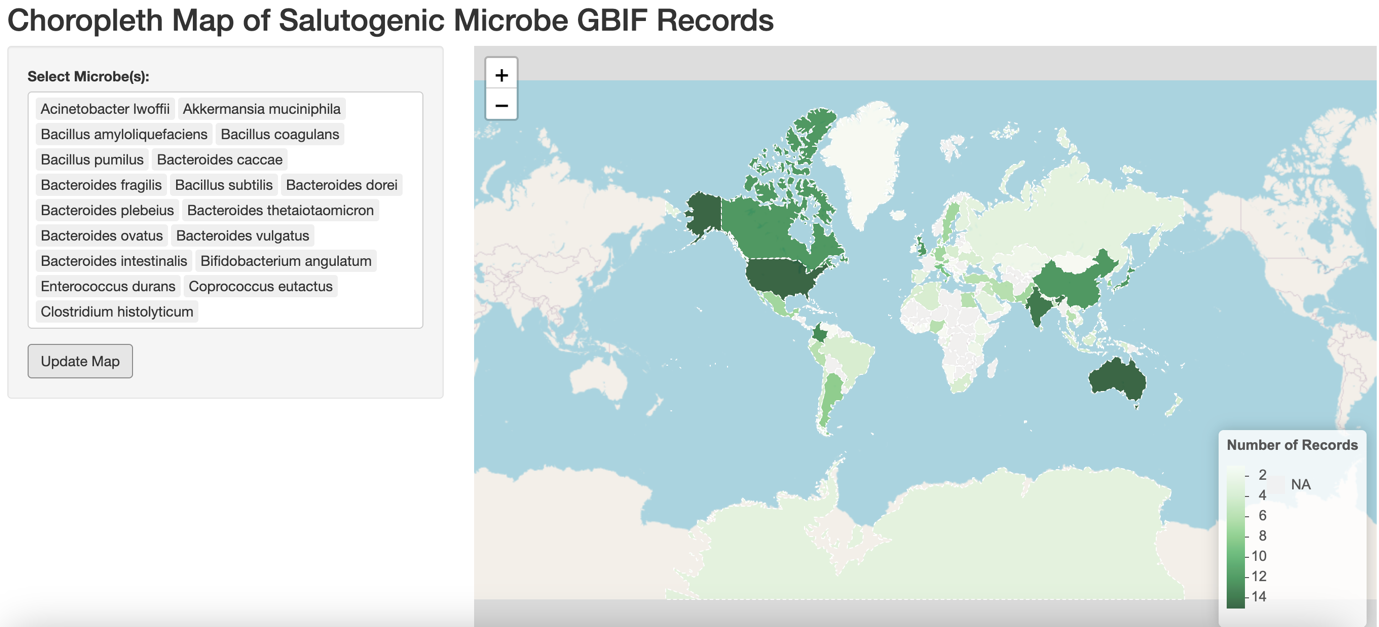
**

Simply select a microbe from the dropdown list and click ‘Update Map’ to view the current global distribution of records, available via GBIF.

This app is under ongoing development and will soon be linked to the *Database of Good Things.*
