# Supplementary material for: Mapping and Cataloguing Microbial and Biochemical Determinants of Health: Towards a ‘Database of Salutogenic Potential’
Source: Microb Biotechnol. 2025 Oct 1;18(10):e70243. doi: 10.1111/1751-7915.70243 (PMC12485223; doi:10.1111/1751-7915.70243)
Supplement: Supplementary file 1 — Appendix S1: mbt270243‐sup‐0001‐AppendixS1.zip. [file MBT2-18-e70243-s001.zip › mbt270243-sup-0001-AppendixS1.docx]

**Methods**

***Identifying salutogenic microbes and compounds***

Search strategy

To identify relevant literature on beneficial microbes and their biochemical products for human health, we performed a systematic search in scientific databases including PubMed, Web of Science and Scopus between 1 October 2024 and 6 October 2024. We searched for articles published between 2000 and 2024 to ensure contemporary relevance, using a combination of keywords and Boolean operators (Figure 1). These were designed to capture studies at the intersection of beneficial microbes, environmental sources and human health outcomes. Our final search string followed a widely used thematic logic: (beneficial microbe terms) AND (environmental terms) AND (health terms). Each thematic group was populated with alternative keywords using the OR operator. For example:

***Beneficial microbes:*** “beneficial microbes” OR “health-promoting microbes” OR “beneficial micro*” OR “probiotic*”

***Environment-related sources:*** “environmental microbiome” OR “environmental microbes” OR “volatile organic compounds” OR “phytoncides” OR “secondary metabolites” OR “essential oil*”

***Health-related outcomes:*** “human health” OR “wellbeing” OR “beneficial to humans” OR “health”

The full query combined these themes using AND logic, ensuring that each retrieved article included at least one term from each category. This approach improved specificity while capturing a broad range of potentially relevant literature. Search terms were adapted for each database’s specific syntax, and additional synonyms were included where appropriate to capture variations in terminology.

Inclusion and exclusion criteria

Information from articles was included if the agent was included in at least two studies and the associated studies: (1) investigated the relationship between environmental microbes or their biochemical products and human health outcomes, (2) explored specific biochemical compounds such as phytoncides, secondary metabolites or other microbially-derived molecules with health-promoting properties, and (3) focused on environmental settings (e.g., urban green spaces, forests, agricultural soils) or nature-based interventions (e.g., forest bathing, horticulture). Articles focusing on harmful biochemical compounds or pathogens were excluded.

Complementary approaches

To complement our database development and literature review, we used the Large Language Model ChatGPT (GPT-4o) as an exploratory tool to assist in identifying candidate beneficial microbial taxa reported in the scientific literature. The goal was not to bypass manual review but to accelerate the initial discovery of potentially relevant taxa by drawing on GPT-4o’s ability to synthesise dispersed information across a broad range of sources. We prompted the model between 3 October 2024 and 7 October 2024 with queries such as: “List all environmental microorganisms with known beneficial health associations in humans.” The resulting taxa were then systematically cross-checked against peer-reviewed literature to verify their validity, with only high-confidence entries retained. Discrepancies or ambiguities were resolved through further examination of the source data. Key information was extracted from the selected articles, including (a) study location and environmental setting, (b) characteristics of the microbial community or biochemical compounds studied, (c) methods used to assess microbiota, biochemical production and human health outcomes, and (d) main findings and implications for health-promoting interventions.

We also cross-referenced our results with the *Optibac* *Probiotics Database* (<https://www.optibacprobiotics.com/professionals/probiotics-database>)––a resource tailored for healthcare professionals, offering in-depth insights into probiotic strains and their associated health benefits. This database provides access to a curated collection of evidence-based information on various probiotic species, strains and their clinical applications.

Identification of beneficial biochemicals

To identify key biochemicals reported to have health benefits, a multi-step approach was used: (1) Database querying, searching for studies that mentioned biochemical compounds such as phytoncides, volatile organic compounds, or secondary metabolites were flagged during the literature search, (2) ChatGPT-assisted synthesis was used to extract and compile information on specific biochemical compounds linked to health outcomes. These results were cross-checked manually against the original studies to ensure accuracy and comprehensiveness. This process included examining the reported mechanisms of action (e.g., immune modulation, stress reduction) and environmental sources of the compounds (e.g., forest environments, soil microbes), and (3) information from an environmental health professional’s toxicology risk assessment database.

DNA accession numbers

To obtain representative DNA accession numbers for microbial taxa included in the *Database of Good Things*, we queried the NCBI Nucleotide database using the Entrez Programming Utilities (E-utilities) via Biopython (v. 1.81). A standardised script was used to search for each taxon name, specified as an [Organism] search field to increase specificity. The search returned the first matching nucleotide entry (retmax=1), from which the accession number was extracted using the efetch function with GenBank format retrieval. A delay of 0.5 seconds was implemented between queries to comply with NCBI rate-limiting policies. Taxon names were first stored in a structured CSV file, and accession results were compiled into an updated dataset. Where no accession number was found or the result was ambiguous, the entry was flagged for manual review. This approach enabled consistent, transparent linkage between taxonomic entries and sequence data for integration into the database (S1).

**Resource description**

***Database design and construction***

The database was constructed (Figure 1) using MySQL Server**^17^**, a relational database management system. A star schema architecture was chosen to optimise query performance and facilitate data analysis. This schema includes (a) a fact table, i.e., a central table containing quantitative data on the relationships between microbes, biochemical compounds and environmental contexts, and (b) dimension tables, i.e., supporting tables providing descriptive metadata on taxa, compounds, environments, locations and sources. Data were imported into SQL Server 2022 Express using SQL Server Management Studio (SSMS)**^17^**. Relationships between tables were established using primary and foreign key constraints to ensure referential integrity. Indexing and normalisation were applied to optimise storage and query efficiency. Queries were developed and tested to validate the schema and ensure accurate retrieval of relationships between microbes, compounds and environmental variables. Following this, data quality checks were conducted to eliminate duplicate entries and correct inconsistencies.

***Visualisation and public accessibility***

To enhance usability and access, the database was integrated with Tableau Public**^18^**. Tableau Desktop was used to establish a connection to the SQL Server database via the SQL Server Connector. Queries were written within Tableau to extract relevant data from the SQL Server instance. Interactive dashboards and visualisations were designed to represent the relationships between microbes, compounds and environmental factors. Filters and parameters were incorporated to allow users to explore specific taxa, compounds or environments of interest. Finalised dashboards were published to Tableau Public (<https://public.tableau.com/app/profile/kate.robinson/viz/SalutogenDashboard/Sheet1?publish=yes>).

***Retrieving biogeographic occurrence data via GBIF and mapping microbial distributions***

To explore the known global distributions of microbial taxa in the *Database of Good Things*, we queried the Global Biodiversity Information Facility (GBIF) using the [pygbif](https://pypi.org/project/pygbif/) Python package (version 0.6.2)**^19^**. GBIF provides open-access biodiversity occurrence data sourced from museum collections, environmental sampling efforts, and citizen science records (S2). We first extracted a unique list of microbial taxon names from the database and passed them to the name_backbone()function to match each input to GBIF's internal taxonomic backbone. This step returned unique taxon keys required for querying occurrence records. For each valid taxon key, we then used the occ_search() function to retrieve all country-level occurrences (up to a maximum of 300 records per taxon, due to API limitations). From these records, we extracted all country names in which the taxon was reported. To reduce false positives, we limited searches to records that had non-null values in the country field. The final output was a CSV file listing each taxon alongside all countries in which it had confirmed GBIF occurrence records. Taxa with no matched GBIF key or no available country-level data were recorded as NA.

To visualise the global distribution of salutogenic microbes, we generated a choropleth map using Plotly (version 5.19.0), an open-source interactive data visualisation library. We transformed the GBIF occurrence data into a format where each country was associated with the number of distinct salutogenic taxa reported in GBIF for that country. The resulting choropleth map was created using plotly.express.choropleth(), colouring each country by taxon richness. Interactive features allowed users to hover over each country to view the specific number of recorded taxa. All data wrangling and processing were performed using pandas (version 2.2.2). Scripts were executed in Python 3.11 via a local Anaconda environment on macOS.

We developed an interactive R Shiny app to visualise the global distribution of microbial taxa using GBIF occurrence data. Country-level records were aggregated by taxon and mapped using ISO3 codes, with spatial layers and visualised via leaflet (<https://jakerobinson.shinyapps.io/salutogen_map-1/>; S3). Users can select taxa of interest to dynamically generate choropleth maps based on record density.

**Results**

**Technical validation**

***User testing, feedback and maintenance***

Beta testing was conducted with a sample of researchers to evaluate functionality and user experience. Feedback was collected and used to refine the database structure and visualisations. The database will be periodically updated to incorporate new research findings, improve functionality and expand its scope. Updates will likely include the addition of new taxa, compounds, environmental contexts and citation information, as well as refinements to visualisation features based on user feedback.

**
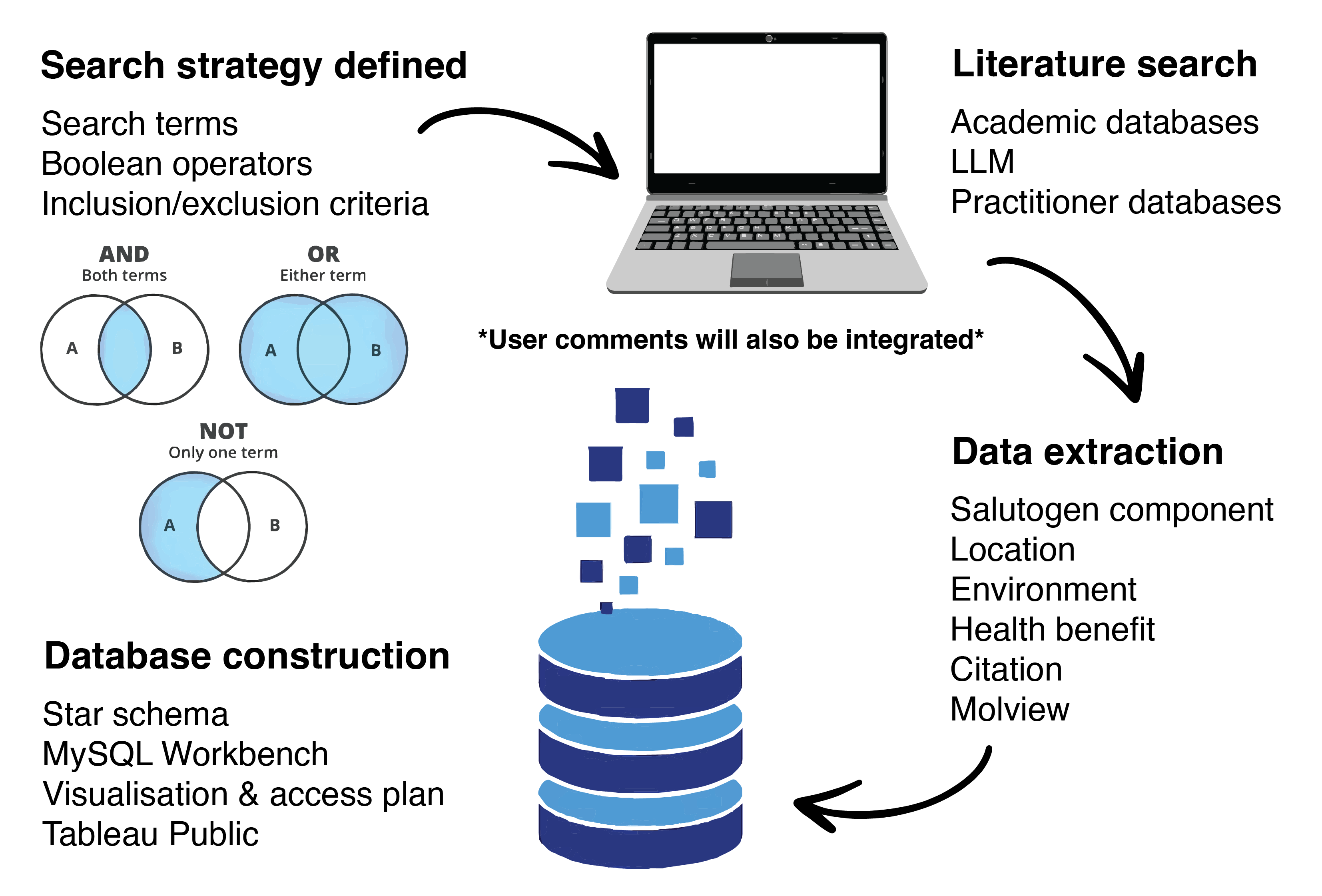
**

**Figure 1.** Workflow for identifying salutogenic components and constructing the relational database.

The *Database of Good Things* comprises two primary categories (with several subcategories), including (1) *Salutogenic microbes* and (2) *Salutogenic biochemical compounds*, and three ‘in-development’ categories, including (1) *Endotoxins* (which offer salutogenic properties via immune training), (2) *mVOCs* (microbial volatile organic compounds), and (3) *Aeronutrients*. The fact table comprises four dimension tables (Health benefit, Classification, Location (e.g., latitude-longitude, South Australia) and Environment (e.g., open eucalypt woodland) (Figure 2). We processed over 300 articles, and 238 were included in the database. In total, we identified 124 potentially beneficial microbial taxa, 14 biochemical compounds and 63 health benefits.


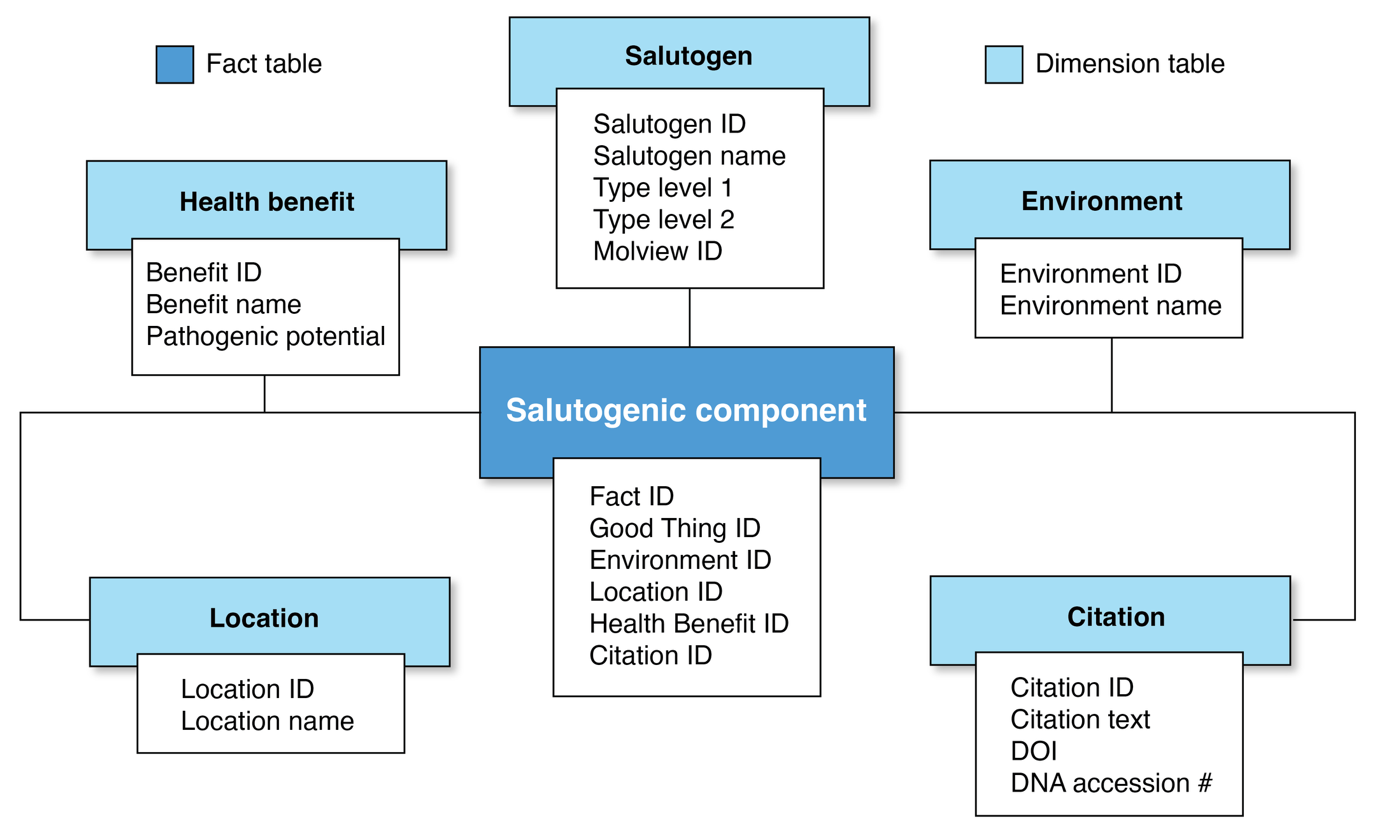


**Figure 2.** Database star schema, including the ‘Salutogenic component’ fact table and dimension tables (i.e., health effect (benefit and, where relevant, pathogenic potential), classification, location and environment, citation).

Following user feedback, we created new classifications for microbial volatile organic compounds (mVOCs), endotoxins and aeronutrients for future exploration. We also created new fields within the dimension tables for pathogenic potential and evidence level and generated Molview URLs for each biochemical compound (e.g., Œ±-Pinene: <https://molview.org/?cid=6654>).

***Grouped themes of purported health benefits***

The potential health benefits associated with the identified salutogenic microbes were grouped into eight broader themes (Figure 3; See Table S1 or the *Database of Good Things* Tableau link for full details: <https://public.tableau.com/app/profile/kate.robinson/viz/SalutogenDashboard/Sheet1?publish=yes> and Table 1 for identified biochemical compounds and potential health benefits).


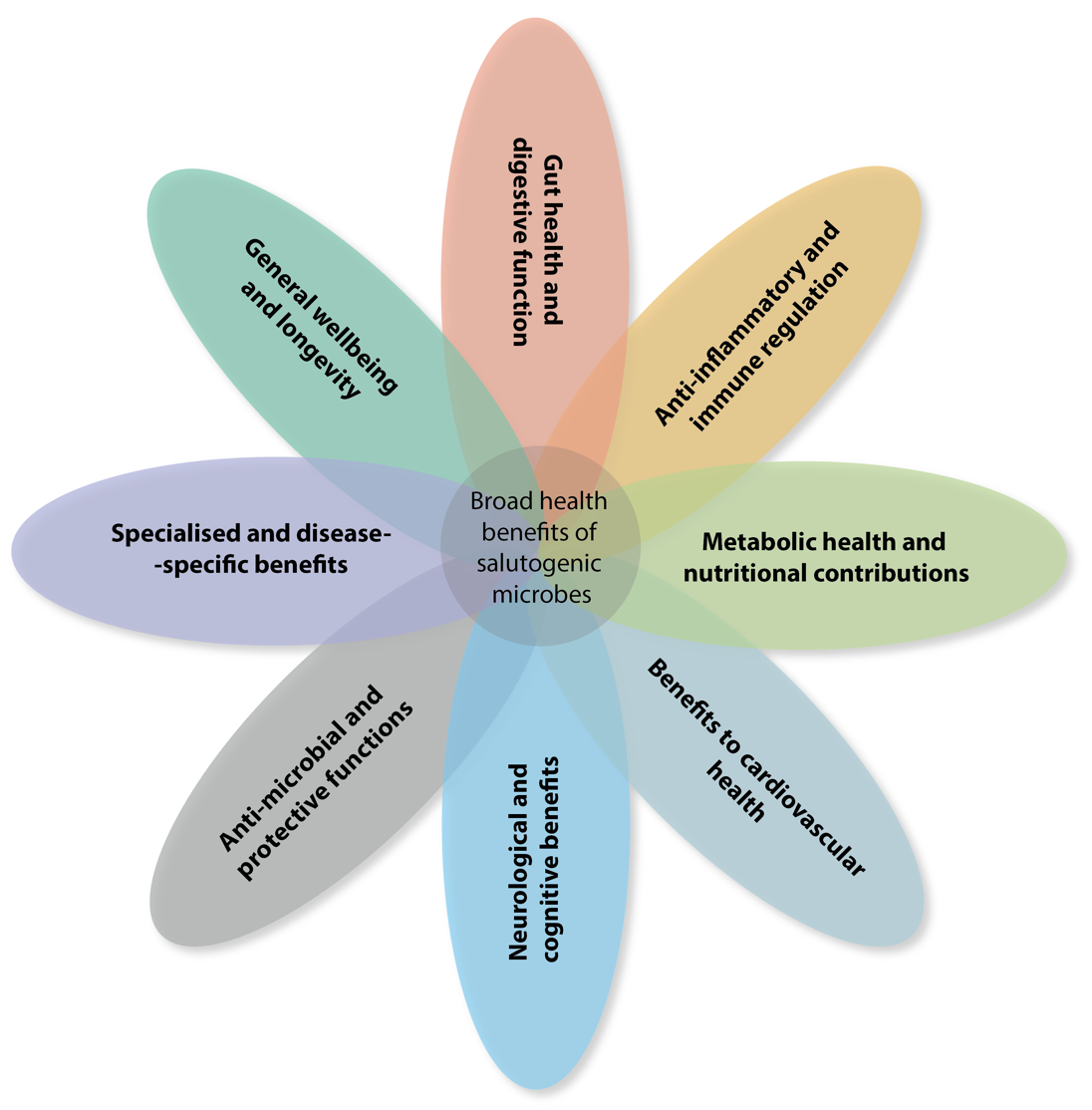


**Figure 3.** Venn diagram of broad (purported) health benefits of the identified salutogenic microbes.

In summary, we identified 12 genera with >2 potentially salutogenic species, including *Lactobacillus, Bacterioides, Bifidobacterium, Bacillus, Ruminococcus, Eubacterium, Roseburia, Lacticasebacillus, Enterococcus, Clostridium, Blautia* and *Parabacteroides* (Figure 4). We identified 42 other taxa with purported salutogenic effects that represent the only species in their respective genus (see the database for further details). We recognise that context is important – for instance, *Bifidobacterium* is often billed as a probiotic, so it would align well in a food or gut context, but not necessarily something one may want to find in HVAC systems in extreme amounts. It would be problematic to categorise a taxon as universally ‘good’ or ‘bad’. Some taxa may confer health benefits in one setting while posing risks in another – hence the inclusion of a ‘pathogenic potential’ dimension (to be populated in future database iterations).


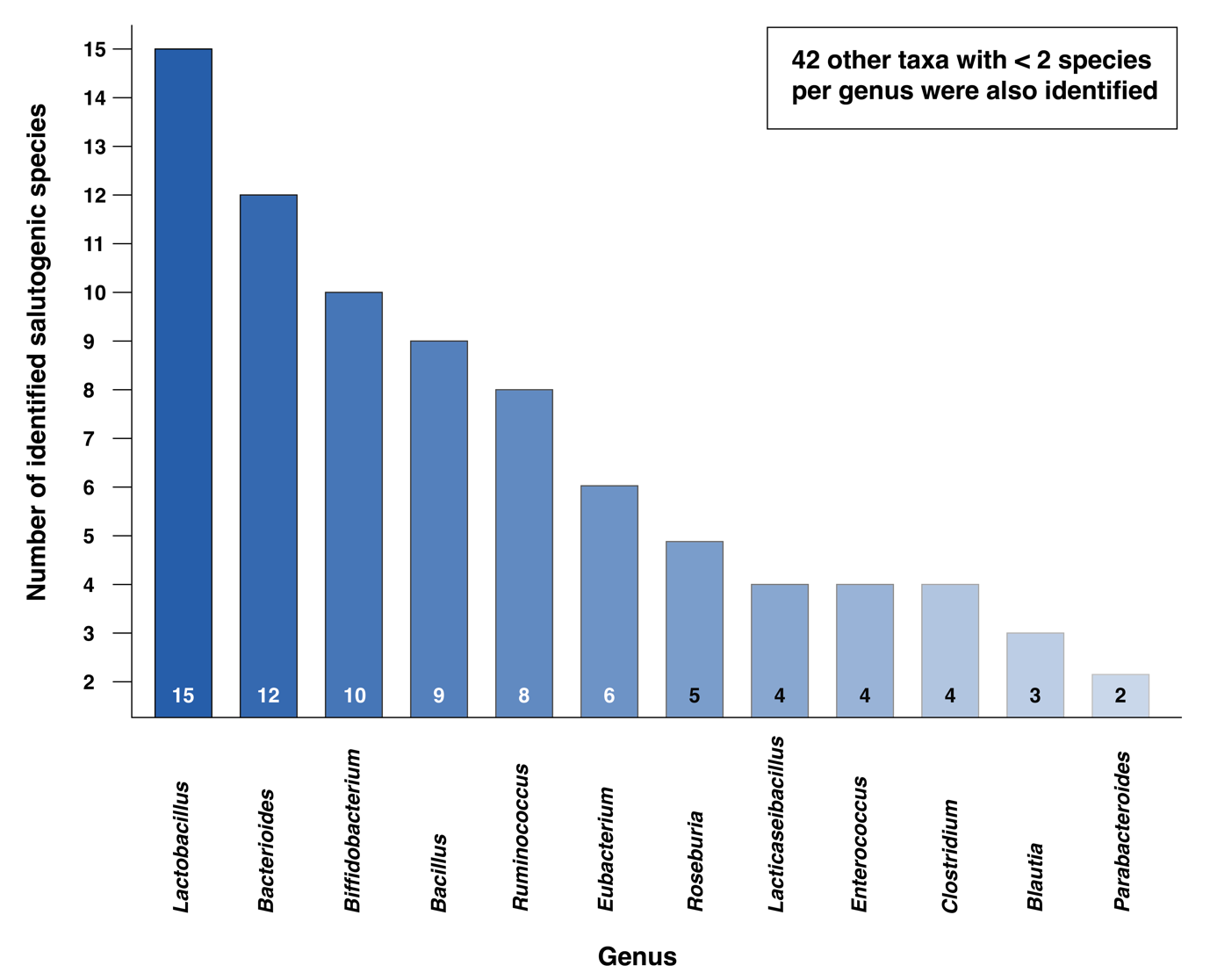


**Figure 4.** Summary of salutogenic microbial taxa at the genus taxonomic level.


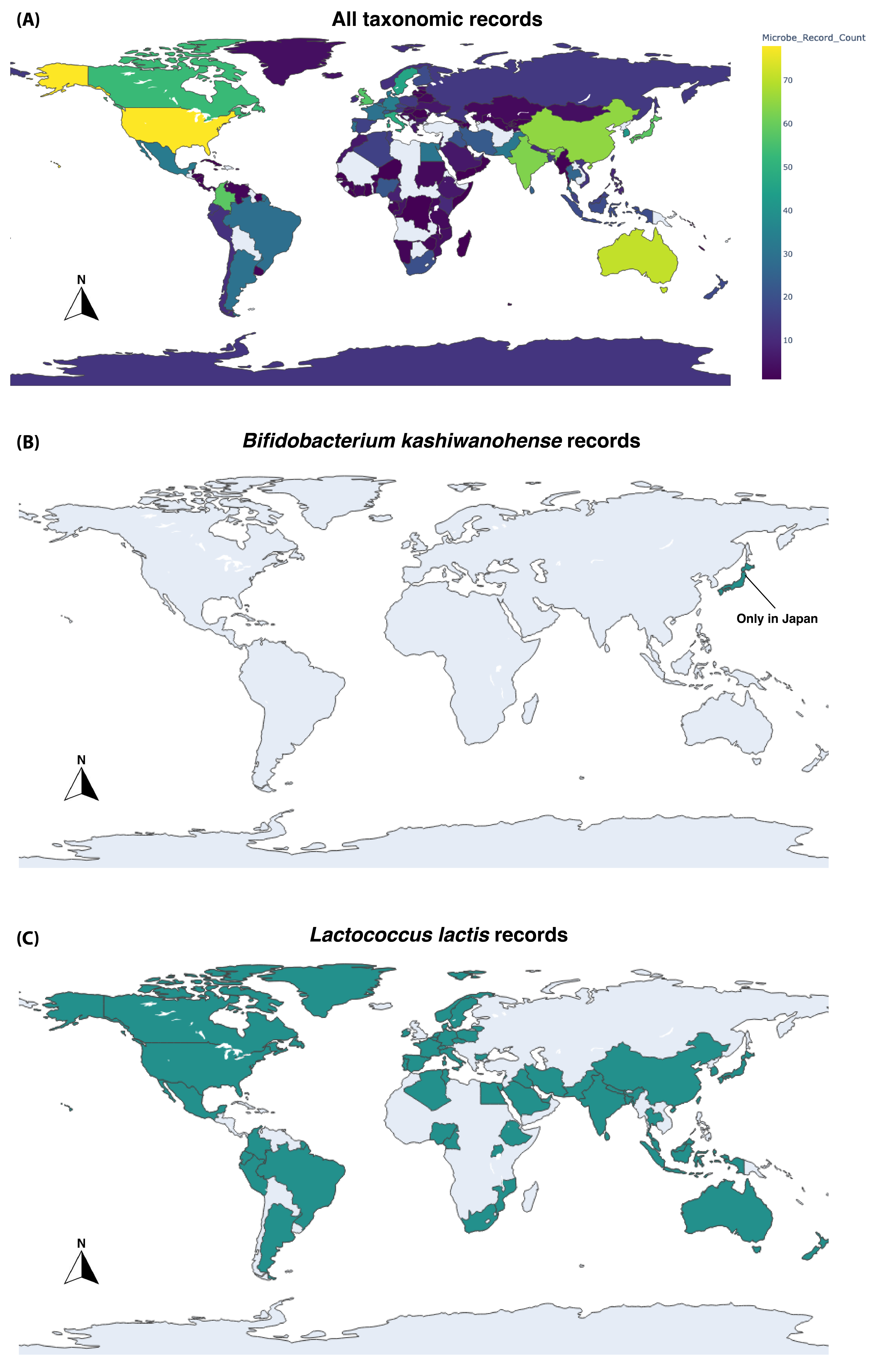


**Figure 5.** Choropleth map showing (A) the number of distinct salutogenic microbial taxa recorded in each country, as reported in the Global Biodiversity Information Facility (GBIF), where colour intensity represents taxon richness, (B) the salutogenic microbe (*Bifidobacterium kashiwanohense*) with the lowest distribution of records, and (C) the salutogenic microbe (*Lactococcus lactis*) with the highest distribution of records. We have an R Shiny App (<https://jakerobinson.shinyapps.io/salutogen_map-1/>), which will soon be connected to the database to allow users to create their own choropleth maps of salutogenic microbe records.

**Table 1.** Biochemical compounds and purported health benefits.

| **Compound** | **Health benefit** | **Citation** |
| --- | --- | --- |
| Œ±-Pinene | Anti-inflammatory, bronchodilator, antimicrobial properties | 20 |
| Œ≤-Pinene | Antimicrobial, anti-inflammatory effects | 20 |
| D-Limonene | Stress reduction, immune system enhancement | 21, 22 |
| Camphene | Antioxidant, antimicrobial activities | 23, 24 |
| Myrcene | Sedative effects, muscle relaxation | 25 |
| Sabinene | Antioxidant, antimicrobial properties | 26, 27 |
| Eucalyptol (1,8-Cineole) | Anti-inflammatory, bronchodilator, antimicrobial, cognitive improvement | 28, 29 |
| Geosmin | Emotional well-being, improved mental state when inhaling fresh soil | 30 |
| Negative ions | Stress reduction, mood improvement, enhanced oxygen intake | 31 |
| Humic acid (airborne particles) | Potential antimicrobial, detoxification roles | 32 |
| Terpenes | Anti-inflammatory, mood enhancement, neuroprotective effects | 33, 34 |
| Nootkatone | Antimicrobial, antifungal, mood enhancement | 35, 36 |
| Linalool | Reduces anxiety, promotes relaxation, improves sleep quality | 37, 3 |
| Borneol | Anti-inflammatory, antimicrobial, neuroprotective effects | 39, 40 |
